# Exercise engagement drives changes in cognition and cardiorespiratory fitness after 8 weeks of aerobic training in sedentary aging adults at risk of cognitive decline

**DOI:** 10.3389/fresc.2022.923141

**Published:** 2022-08-02

**Authors:** Carrie A. Hinchman, Danylo F. Cabral, Marissa Ciesla, Marti Flothmann, Christina Nunez, Jordyn Rice, David A. Loewenstein, Marcela Kitaigorodsky, Lawrence P. Cahalin, Tatjana Rundek, Alvaro Pascual-Leone, Gabriele Cattaneo, Joyce Gomes-Osman

**Affiliations:** ^1^School of Medicine, New York Medical College, Valhalla, NY, United States; ^2^Department of Physical Therapy, University of Miami Miller School of Medicine, Coral Gables, FL, United States; ^3^Linus Health, Waltham, MA, United States; ^4^Department of Neurology, University of Miami Miller School of Medicine, Miami, FL, United States; ^5^Evelyn McKnight Brain Institute, University of Miami Miller School of Medicine, Miami, FL, United States; ^6^Center for Cognitive Neuroscience and Aging, University of Miami Miller School of Medicine, Miami, FL, United States; ^7^Department of Neurology, Harvard Medical School, Boston, MA, United States; ^8^Hinda and Arthur Marcus Institute for Aging Research, Hebrew SeniorLife, Rosindale, MA, United States; ^9^Deanna and Sidney Wolk Center for Memory Health, Hebrew SeniorLife, Rosindale, MA, United States; ^10^Guttmann Brain Health Institute, Barcelona, Spain; ^11^August Pi i Sunyer Biomedical Research Institute, Barcelona, Spain

**Keywords:** aerobic exercise, cognitive enhancement, cardiorespiratory fitness, exercise engagement, aging adults

## Abstract

**Background:**

With our aging population, many individuals are at risk of developing age-related cognitive decline. Physical exercise has been demonstrated to enhance cognitive performance in aging adults. This study examined the effects of 8 weeks of aerobic exercise on cognitive performance and cardiorespiratory fitness in sedentary aging adults at risk for cognitive decline.

**Methods:**

Fifty-two participants (age 62.9 ± 6.8, 76.9% female) engaged in eight weeks of moderate-to high-intensity exercise (19 in-person, 33 remotely). Global cognition was measured by the Repeatable Battery for the Assessment of Neuropsychological Status, the Delis-Kaplan Executive Function System, and the Digit Span subtest of the Wechsler Adult Intelligence Scale (WAIS) Fourth Edition. Cardiorespiratory fitness was measured *via* heart rate recovery at minute 1 (HRR1) and 2 (HRR2), and exercise engagement (defined as percent of total exercise time spent in the prescribed heart rate zone). We measured pre and post changes using paired *t*-tests and mixed effects models, and investigated the association between cardiorespiratory and cognitive performance using multiple regression models. Cohen's d were calculated to estimate effect sizes.

**Results:**

Overall, 63.4 % of participants demonstrated high engagement (≥ 70% total exercise time spent in the prescribed heart rate zone). There were significant pre-post improvements in verbal fluency and verbal memory, and a significant decrement in working memory, but these were associated with small effect sizes (Cohen's d <0.5). Concerning cardiorespiratory fitness, there was a pre-to-post significant improvement in HRR1 (*p* = 0.01, d = 0.30) and HRR2 (*p* < 0.001, d = 0.50). Multiple regressions revealed significant associations between cardiorespiratory and cognitive performance, but all were associated with small effect sizes (Cohen's d < 0.5). Interestingly, there were significant between-group differences in exercise engagement (all *p* < 0.001), with remote participants demonstrating greater exercise engagement than in-person participants.

**Conclusion:**

Improvements in cognition and cardiorespiratory fitness were observed after 8 weeks of moderate to high-intensity exercise in aging adults. These results suggest that committing to a regular exercise regimen, even for a brief two-month period, can promote improvements in both cardiorespiratory fitness and cognitive performance, and that improvements are driven by exercise engagement.

## Introduction

Our global population is aging, with individuals 65 and older projected to make up 30% of the population over the next 30 years (1). As individuals live longer, many will develop cognitive decline, which, despite great strides in the field, is still lacking effective preventions and treatments (1, 2). Cognitive decline not only affects those who are afflicted with it, but also their loved ones who become caregivers, and the healthcare system that they rely on. Therefore, the development of successful strategies to combat cognitive decline and preserve individuals' abilities to function independently and maintain fulfillment is vital, for patients, their loved ones, and for society as a whole (3, 4).

Taking active steps toward a healthier lifestyle can have a profound impact on brain health among aging individuals. Modifiable factors, such as pursuing a healthier diet, having adequate sleep, keeping an active mind, nurturing social interactions, and practicing regular exercise can decrease dementia risk by as much as 40% (5–8). Exercise particularly, may be a powerful approach for maintaining brain health in aging. Being more physically active is a high health-related priority for most individuals in older age (9). Practicing regular exercise can improve overall health, and help older adults maintain physical function and independence. Importantly, a wealth of strong scientific evidence supports cognitive improvement in aging adults who are physically active (7, 10, 11). An encouraging finding is that the exercise types backed up by such evidence are low cost, such as walking, or jogging.

Notwithstanding, older adults find it difficult to commit to an active lifestyle. At present, only a third of older adults globally adhere to the recommended exercise guidelines of 150 min of moderate (or 75 min of vigorous) exercise each week (12). Nonetheless, and importantly, most people are motivated to improve their lifestyle, especially if by doing so they will meaningfully improve their brain health. In a recent survey conducted by the Global Brain Health Survey (3), 70% of respondents indicated that memory problems would be a key motivator for them to improve their lifestyle. An important caveat, however, is that their decision was contingent on the effectiveness of this lifestyle change to improve their cognition.

While at a group level, exercise is effective for improving cognition in older adults, the variability in cognitive response to exercise remains an important barrier to advancing knowledge in this field. Some individuals show robust cognitive benefits from exercise, while others show less pronounced improvements (13, 14). In addition, not all cognitive abilities appear to be equally influenced by exercise. For example, most exercise-induced improvements are reported in the areas of executive functioning and processing speed, when compared to other cognitive abilities such as memory and global cognition (5, 14, 15). This variability is a barrier to advancing knowledge on the specific exercise programs or dosages that are most beneficial to support cognitive brain health in aging and addressing this barrier will require carefully examining the mechanisms responsible for exercise-induced cognitive gains.

Gains in cardiorespiratory function are important drivers of the cognitive improvements that occur in response to aerobic exercise (4, 12, 16). The cardiorespiratory fitness (CRF) hypothesis incorporates cardiovascular health with neurological mechanisms and postulates that physiological changes resulting from exercise lead to improved cognitive function (17). This hypothesis proposes that increased cerebral blood flow and perfusion result in greater oxygenation and glucose transport to the brain, leading to increased expression of vascular endothelial growth factor and cerebral angiogenesis, increased expression of neurotrophic factors such as brain-derived growth factor, and increased gray and white matter volume (17–20). At the brain level, these cardiorespiratory/cardiovascular gains improve cerebrovascular function, and promote neuroplasticity, ultimately leading to more efficient neural processing (21–23). Importantly, it is suggested that there may be a dose-response relationship between exercise and health outcomes such as cardiorespiratory fitness and cognition. Studies have shown that greater benefits may be achieved with greater amounts of activity, as well as by taking part in more vigorous activity (24). It is therefore conceivable that individuals who are motivated to maintain more vigorous exercise regimens may see greater health benefits than their peers who engage in less intense exercise, or do so less frequently.

Maximal oxygen consumption during a progressive exercise (VO2peak) is the gold-standard outcome measure for cardiorespiratory gains and is widely used in this field (5, 25). One shortcoming of this approach, however, is that VO2peak assessments are lengthy and require sophisticated equipment and specialized personnel for administration and interpretation. To further advance knowledge in this field, we need measures that can be administered more rapidly and easily, and ideally in most settings where older adults are currently exercising (clinics, gyms, community centers, and at home). The challenges in committing to an active lifestyle highlight the need to improve our development of metrics of success that can empower and support individuals to take a more active role in carrying out the lifestyle changes that they wish to pursue.

Heart rate reserve (HRR) is an established outcome measure of cardiovascular function that has been mostly investigated in individuals with heart failure (26), that requires only a few minutes and access to a heart rate monitor. HRR assesses autonomic mechanisms involved in the exercise response and is an independent predictor of a future cardiovascular event and all-cause mortality, and is strongly associated with cognitive impairment (27–29). However, HRR has not been previously employed to investigate exercise-induced cognitive benefits in older adults. If found useful, HRR could greatly advance knowledge in this field because it may be a helpful biomarker of effective exercise in relation to cognitive function. In addition, HRR is easy to monitor and it can be administered at the end of exercise regimens, thus, being a good candidate to study an exercise dose-response.

The main objective of the present study was to identify clinically meaningful effects of aerobic exercise on cardiorespiratory and cognitive performance. This research may be relevant for future studies aimed at optimizing exercise interventions for cognitive gains in aging. We conducted a single-arm intervention trial estimating the degree to which 8 weeks of moderate to high intensity aerobic exercise would induce within-participant change in cardiorespiratory and cognitive performance in adults of age 55 years or older. We hypothesized that cardiorespiratory and cognitive performance would improve from baseline. We further hypothesized that exercise engagement (defined as time spent in target heart rate zone) would be a modifier of cardiorespiratory and cognitive effects.

## Methods

This study was approved by the Institutional Review Board at the University of Miami Miller School of Medicine and all participants provided written informed consent. This study was registered on clinicaltrials.gov (NCT03804528). The detailed study protocol and adaptation methods of measures and intervention has been previously published (30). In this study, the focus is on the primary measures of cognitive function, cardiorespiratory fitness and exercise adherence.

Briefly, participants completed a battery of cognitive and cardiorespiratory fitness assessments prior to and following the 2-month exercise intervention. All assessments were conducted by a study member who received specific training for each test. Approximately halfway through enrollment, the protocol was adapted to accommodate public health restrictions imposed by the COVID-19 pandemic. As a result, the study was adapted to be home-based, with remotely delivered assessments and exercise, using standardized telehealth procedures, while maximizing the collection of outcome measures and aiming to match the dose and effort of the exercise intervention (30). Participants were sent a study kit, containing the necessary equipment for their assessments and exercise sessions. A familiarization session was scheduled through the Zoom platform and participants received detailed information on procedures and correct equipment functioning. Participants also received a study booklet containing specific recommendations on how to follow the study procedures, set up the study devices, and prepare for the assessment, definitions of important terms, and daily exercise sheets to be completed during the exercise sessions. The supervised in-person exercise intervention was from February 2019 to March 2020, and the home-based remotely supervised exercise intervention was from August 2020 to April 2021.

### Participants

In-person participants were recruited from the university community by flyers posted on the medical school campuses, and the greater Miami community by posting flyers in public libraries. In addition, participants were recruited online using ResearchMatch.org and by identifying potential participants from the University Research Informatics Data Environment *via* the Consent to Contact Initiative. For the remote participants, during COVID-related restrictions, we additionally posted flyers on social media and disseminated the study *via* a neighborhood app (Next Door). Interested individuals were invited to an in-person or virtually *via* Zoom for Healthcare (Zoom Video Communications Inc) screening visit to collect physical measures (e.g., vital signs), demographics, and medical and family history.

Inclusion criteria included individuals aged 55 years and older, who were sedentary (as determined by the short version of the International Physical Activity Questionnaire [IPAQ]), without clinically detectable cognitive impairment (Montreal Cognitive Assessment score ≥24) and speaking English as a primary language. Participants were considered at risk of cognitive decline due to their age, sedentary activity, and other lifestyle factors known to contribute to cognitive decline. An additional inclusion criterion for the remote participants was having basic computer skills (accessing an email or using the internet). Exclusion criteria included any unstable medical condition (i.e., uncontrolled hypertension), medical contraindication to physical exercise, or contraindications for any of the assessment methods.

### Neuropsychological test battery

In this manuscript, we report on neuropsychological assessments that were equivalent in both in-person and remote participants. The adaptation to a virtual administration of this test was determined according to internal guidelines set forth by the Neuropsychology department at the University of Miami. Some tests were modified to accommodate a remote testing procedure, as noted below. Alternate versions of each test were used at baseline and follow-up testing visits in an attempt to mitigate practice effects.

#### Repeatable battery for the assessment of neuropsychological status

The RBANS is a brief battery used to measure an individual's cognitive state (31). It consists of 12 subtests that are used to calculate index scores for five cognitive domains: immediate memory, visuospatial/constructional, language, attention, and delayed memory. The subtests are then summarized to obtain a total score for global cognition. In the virtual test administration, it was not possible to conduct the Coding and Figure Recall subtests. Data imputation was used to account for these tests in the statistical analysis. For the Figure Copy subtest, as per our instructions, we held up the figure to the screen, allowing the participant to view and reproduce the drawing. They had up to 4 min to complete this task. When their time was up (or if they had indicated completion), we asked them to hold their drawing up to the screen. We took a picture of the drawing, and performed the grading offline.

#### Delis-Kaplan executive function system

One secondary endpoint was the Verbal Fluency subtest of the DKEFS used to assess categorical verbal fluency (32). The Verbal Fluency subtest of the DKEFS is a widely used measure that assesses phonemic fluency by asking individuals to generate as many words that begin with a certain letter for the alphabet for three trials of different letters, and semantic fluency by asking individuals to name as many items as they can in each of two categories. Score is determined based on the number of letters and unique items produced per category. DKEFS also assesses set-shifting using a subtest where individuals are asked to generate words switching between two categories (i.e.: a musical instrument and a piece of fruit). Score is determined by the total number of correct switches between categories, and the total number of correct responses given.

#### Digit span subtest of the wechsler adult intelligence scale-fourth edition

The Digit Span subtest of the WAIS-IV was used to assess attention and memory (33). This measure consists of three parts: Digit Span Forward (DSF), Digit Span Backward (DSB), and Digit Span Sequencing (DSS). Participants are read increasingly longer lists of numbers (starting with two digits and increasing in difficulty up to nine digits) and are asked to repeat them verbatim, in backward order, and sequentially (i.e., from smallest to largest) on DSF, DSB and DSS respectively.

### Cardiorespiratory fitness testing

HRR was selected as the primary measure of cardiorespiratory fitness. Briefly, the individual undergoes an exercise test, and the deceleration of the heart rate after exercise cessation is quantified. HRR is defined as the change in the heart rate from the peak of exercise to the heart rate after 1-min (HRR-1) and 2-min test cessation (HRR-2) (26). HRR measures were collected following an exercise test (in-person participants, the Incremental Shuttle Walking Test [ISWT] (34, 35) and remote participants the 1-Min Sit-to-Stand Test [1-STS]), given that the study team decided it was not feasible to perform the ISWT remotely (36). Specific procedures of both tests are described in the published protocol (30). Prior to the test, participants were screened for signs and symptoms that would contraindicate exercise (e.g., dizziness, nausea, chest pain). Participants were fitted with a heart rate monitor (Polar H10, Polar Electro Inc), seated in a comfortable chair for 5 min, and blood pressure, heart rate, rate of perceived effort, and oxygen saturation were measured and documented at rest, during, upon completion, and 5 min after test cessation. Aerobic capacity measures were the walking distance from the ISWT and the maximal sit-to-stand repetitions completed in 1 min for the 1-STS.

### Study adherence and engagement

#### Study adherence

The adherence rate was estimated using the following variables: (1) proportion of participants who completed the planned intervention, and (2) total number of days to complete the intervention from exercise session 1 to session 24.

#### Engagement: Time spent in target heart rate zone

We calculated the proportion of time spent within the prescribed target heart zone for each session (24 in total) for each participant and averaged it across the participants in both in-person and remote exercise participants. This was meant to give insights into the extent to which participants adhered to the supervised exercise prescription.

### Exercise intervention

All participants engaged in 1 h of supervised exercise, three times a week for eight consecutive weeks (a total of 24 sessions). Participants were offered the opportunity to make up missed sessions. Exercise sessions consisted of a 5-min warm-up, 50 min of exercise in the prescribed target heart rate zone (described below), and a 5 min cool-down. Participants underwent continuous heart rate monitoring throughout the exercise sessions using the heart rate monitor. Blood pressure was measured with participants seated for 5 min prior to the session and after completing the session.

In-person participants completed their supervised exercise intervention at the University of Miami Miller School of Medicine Wellness Center. Participants were fitted with a heart rate monitor and were instructed to exercise to maintain 55–64% (moderate intensity) of their maximal heart rate (determined by the fitness testing) during weeks 1–4, and to maintain 65–90% (high intensity) of their maximal heart rate during weeks 5–8. Participants were always supervised by a member of the study team, and the protocol was overseen by the PI, a licensed physical therapist. Participants could select 1 of the 4 available exercise modalities (treadmill, elliptical, stationary bike, or stationary recumbent bike) at each session. Participants were offered breaks to rest and were encouraged to hydrate throughout the session. During the sessions, participant's heart rate and exerted effort, measured using the Borg scale, were assessed every 5 min of the 50-min session and 5 min after the end of the session. The Borg scale is a numerical scale with scores ranging from 6 to 20, where 6 represents rest (no effort), and 20 represents maximal effort (37).

Remote participants took part in a supervised home-based exercise intervention over Zoom using standard telehealth procedures. Participants received a pre-planned routine of alternating body-weight exercises developed by the study team, which included exercises such as squats, marching in place, and wall push-ups, among others. Intervention details are provided in the published protocol (30). Participants were monitored utilizing the same methods and verbal prompts as the in-person participants, and were instructed to maintain the same levels of effort as during in-person participants (i.e., 55–64% during weeks 1–4 and 65–90% during weeks 5–8). In summary, the intervention was structured in warm-up, five 10-min timed blocks consisting of 8 min of exercise and 2 min of rest completing a total of 10 unique exercises, followed by a cool down.

### Statistical methods

All statistical analyses were performed using IBM SPSS Statistics for Macintosh (v. 28, IMB Corp, USA) and StataCorp 2021 (v. 17, StataCorp LLC, USA) using a two-tailed 95% confidence interval (α = 0.05). All data entries were coded and double-entered into an Excel (Microsoft Corp) spreadsheet for analysis. Data are presented as means ± SD for continuous variables and as frequency and percentage (%) for categorical variables. To test the homoscedasticity assumption, we used the default test in the Stata Breusch-Pagan/Cook-Weisberg test. To test the normality of the studentized residuals, we visually inspected using the Q-Q plots and tested using the Shapiro-Wilks test of normality. We compared in-person and remote participants with regards to demographic and health status (Table 1) using pooled *t*-test for continuous variables and Chi-square test for categorical variables.

**Table 1:** Demographics and global health status.

	**All participants** **(*n =* 52)**	**In-person** **(*n =* 19)**	**Remote** **(*n =* 33)**	***p*** **-value**
**Demographics**				
Age, years	62.9 ± 6.8	61.8 ± 7.1	63.5 ± 6.6	0.38
Age, range	56–87	56–87	56–79	
**Gender**, ***n (%)***				
Female	40 (76.9)	12 (63.2)	28 (84.8)	0.08
**Ethnicity**, ***n (%)***				
Black	7 (13.5)	2 (10.5)	5 (15.1)	0.02^a^
White	24 (46.1)	4 (21.1)	20 (60.6)	
Hispanic	17 (32.7)	10 (52.6)	7 (21.2)	
Hispanic/White	3 (5.8)	2 (10.5)	1 (3.1)	
Asian	1 (1.9)	1 (5.3)	0 (0)	
**BMI**, ***n (%)***				
Normal (18,5–24.9)	14 (26.9)	3 (15.8)	11 (33.3)	0.37
Overweight (25–29.9)	19 (36.5)	7 (36.8)	12 (36.4)	
Obese Class 1 (30–34.9)	13 (25.0)	7 (36.8)	6 (18.2)	
Obese Class 2 (35–39.9)	6 (11.5)	2 (10.5)	4 (12.1)	
Obese Class 3 (> 40)	0 (0.0)	0 (0)	0 (0)	
**BMI**, *mean ± SD, kg/m^2^*	28.1 ± 5.1	29.5 ± 4.9	27.3 ± 5.1	0.13
**Education level**, ***n (%)***				
High school	9 (17.3)	3 (15.8)	6 (18.2)	0.80
Undergraduate/Associate degree	27 (51.9)	11 (57.9)	16 (48.5)	
Graduate degree	16 (30.8)	5 (26.3)	11 (33.3)	
**Health status**				
Global cognition, MoCA total, mean ± SD	26.4 ± 1.9	25.8 ± 1.9	26.8 ± 1.9	0.09
Hospitalized, *n (%)*	41 (78.8)	13 (68.4)	28 (84.8)	0.17
Taking prescribed medication, *n (%)*	43 (82.7)	15 (78.9)	28 (84.8)	0.59
Smoking history, *n (%)*	13 (25.0)	2 (10.5)	11 (33.3)	0.06
Alcohol consumption*, n (%)*	41 (78.8)	13 (68.4)	28 (84.8)	0.17
Caffeine consumption, *n (%)*	49 (94.2)	17 (89.5)	32 (96.9)	0.27
Current diseases and comorbidities, mean ± SD range (n)	2.8 ± 2.0 0 - 9	3.6 ± 2.5 0 - 9	2.4 ± 1.5 0 - 5	0.07
Hypertension, *n (%)*	25 (48.1)	13 (68.4)	12 (36.4)	0.17
Under beta-blocker, *n (%)*	6 (11.5)	1 (5.3)	5 (15.1)	0.26
Arthritis or joint pain, *n (%)*	22 (42.3)	9 (47.4)	13 (39.4)	0.57
Depression/Anxiety, *n (%)*	13 (25.0)	3 (15.8)	10 (30.3)	0.23
Thyroid disease, *n (%)*	12 (23.1)	4 (21.0)	8 (24.2)	0.79
Lung/Asthma disease, *n (%)*	11 (21.1)	5 (26.3)	6 (18.8)	0.49
Tumor or cancer, *n (%)*	10 (19.2)	6 (31.6)	4 (12.1)	0.09
Migraine/Severe headache, *n (%)*	9 (17.3)	4 (21.1)	5 (15.1)	0.59
Heart disease, *n (%)*	8 (15.4)	5 (26.3)	3 (9.1)	0.10
Hearing loss, *n (%)*	6 (11.5)	4 (21.1)	2 (6.1)	0.11
Fainting/Dizzy spells, *n (%)*	6 (11.5)	4 (21.1)	2 (6.1)	0.11
Stomach/Intestinal disease, *n (%)*	4 (7.7)	1 (5.3)	3 (9.1)	0.61
Diabetes, *n (%)*	4 (7.7)	3 (15.8)	1 (3.0)	0.10

*BMI, Body Mass Index; MoCA, Montreal Cognitive Assessment*.

a *Statistical significance (p < 0.05)*.

To test our primary hypothesis, we compared the cognitive performance and cardiorespiratory fitness measures before and after the 8-week exercise intervention using paired *t-*tests. With a significance level of 0.05, our sample of 52 participants provided a two-tailed 99% power to detect at least a moderate Cohen's d effect size of 0.67 in pre-to-post change in global cognition as assessed by the RBANS total score and in Group(engagement)^*^Time interaction effect as assessed by z-score global cognition. In addition, our sample provided 94% power to detect at least a moderate effect size of 0.50 in pre-to-post change in cardiorespiratory fitness as assessed by HRR2.

Neuropsychological tests were clustered in cognitive domains using a principal component analysis. Pre and post cognitive raw scores on individual tests were Z-score normalized before their inclusion in the principal component analysis with Oblimin rotation, considering the probable correlation between latent factors (38). Level of factor loading was set at 0.30. Cognitive domains were then created as the composite average of the Z-scores for each test score per the results from the principal component analysis. The global cognition score represents the average of all z-scores of all cognitive assessments.

Engagement (time spent in the target heart rate zone) is likely a major confounder of the effect of the exercise intervention, given that cardiorespiratory improvements have been demonstrated to drive cognitive improvements (14). To address this aspect as part of our secondary hypothesis, we first conducted additional analyses to evaluate its influence pre-post effects on cardiorespiratory fitness and cognition. We first quantified the proportion of total intervention time that participants maintained their prescribed target heart rate zone. The mean exercise engagement was 76.7% (95%Ci 73.9–79.6). The cutoff point of 70% was chosen to separate low-to-moderate engagement participants from high engagement participants, as it was the nearest tenth number to the lower limit of the confidence interval. Dichotomous exercise engagement variables were created, and participants were subdivided into two groups: those who maintained the prescribed target heart rate more than 70% of the exercise time (high engagement) and those who maintained the prescribed target heart rate less than 69.9% of the exercise time (low-to-moderate engagement). To assess if there was an interaction effect between the two subgroups (high and low engagement), standardized cognitive domains, overall global scores and cardiorespiratory fitness measures (HRR1, HRR2, and aerobic capacity) data were entered into mixed-effects linear models with a main effect of *Time* (pre vs. post), *Group* (high vs. low-to-moderate engagement), and Group^*^Time interaction effect. An exploratory analysis compared exercise engagement between in-person and remote participants using independent *t*-tests.

To additionally address another aspect of our secondary hypothesis regarding the association between effects on cardiorespiratory fitness and cognition, while accounting for differences in engagement, multiple linear regressions were fitted to analyze the association between cardiorespiratory fitness measures (HRR1, HRR2, and aerobic capacity [in-person and remote participant's aerobic capacity were z-score normalized]) and standardized cognitive domains. Then, models were adjusted to control for engagement (two subgroups, low and high), as well as baseline performance in cognition and cardiorespiratory fitness. Regression models were inspected to avoid departure from collinearity using the variance inflation factor assuming mean VIF < 10.

Multiple comparisons were corrected for using Benjamini and Hochberg's false discovery rate, at a *q* value of 0.05, after pooling the *P* values from the baseline comparisons with *t-*test or Chi-square test, linear mixed-effect models, and regression analyses for each predictor model. In specific instances when data was missing in individuals who completed the intervention, we employed random data imputation. We used systematic data imputation to address the missing values in the RBANS (Coding and Figure Recall), which was not available for those who participated in the intervention remotely. Effect sizes were interpreted based on published values: <0.2 trivial effect, 0.2–0.5 small effect, 0.5–0.8 moderate effect, >0.8 large effect (39).

## Results

### Participants and study adherence

**Figure 1:**
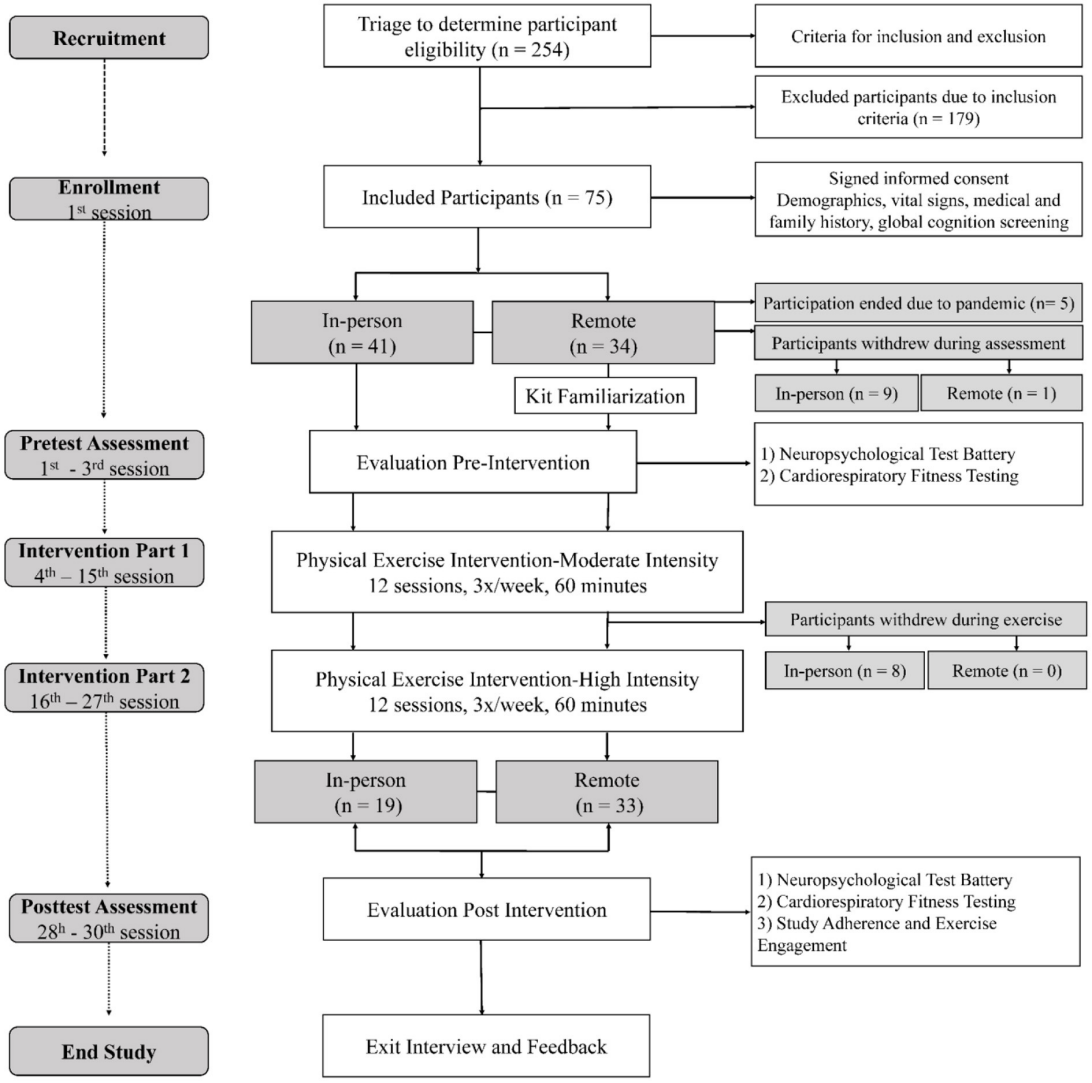
Study flowchart.

Seventy-five participants were enrolled into the study, and 52 (69.3%) completed the study (Figure 1). Forty-one participants were enrolled in in-person exercises and 34 participants were enrolled in remote exercises. Of the 41 subjects enrolled in in-person exercises, 19 (46.3%) completed the study. Nine participants withdrew during assessment sessions and eight participants withdrew during exercise intervention due to time commitment (*n* = 10), health reasons (*n* = 5), and a medical contraindication to exercise that occurred after study onset (*n* = 2). Five participants were enrolled in the study just before the COVID-19 pandemic, and their participation was ended due to resulting public health restrictions. All but one of the 34 participants (97.1%) enrolled in remote exercises completed the study; one participant withdrew during the assessment due to time commitment. The average time (days) of exercise was 60.5 (±7.8); 64.4 days (±9.2) for in-person exercises and 58.2 (±5.9) for remote exercises.

As summarized in Table 1, participants who completed the study were 62.9 ± 6.8 years old (vs. 62.0 ± 5.1 age of those who did not complete the study), and the majority were female (76.9%). The majority of participants were from White and Hispanic race-ethnic groups, overweight, and highly educated. On average, participants reported approximately 3 current diseases or comorbidities, with hypertension being present in 50%. In-person and remote participants of the study were similar, except for the difference in race-ethnicity (*p* = 0.02). Thus, all remaining analyses were conducted by combining all 52 participants from both in-person and remote exercise participants.

### Cognitive function

The principal component analysis indicated the presence of 6 principal components for the cognitive scores. The first factor reflected verbal fluency and language and included the RBANS subsets of semantic fluency (0.794) and picture naming (0.565), and DKEFS subsets of letter fluency (0.781) and category fluency (0.660). The second component reflected verbal memory and comprised RBANS subsets of list learning (0.838), list recall (0.815), story memory (0.778), story recall (0.747), and list recognition (0.723). The third component of set-shifting contained the DKEFS subsets of category switching (−0.916) and category switching accuracy (-0.909). Working Memory was reflected in the fourth domain and included all 3 subsets of the Digit Span (forward = −0.872, backward = −0.797, sequence = −0.574), and the RBANS digit span subset (−0.793). The fifth component included RBANS subset of figure recall (0.748) and coding (0.719) reflecting visual memory. Lastly, the sixth component reflected visuospatial abilities and contained the RBANS subsets of figure copy (0.808) and line orientation (0.727).

Table 2 summarizes the results for all participants. Pre-to-post exercise analyses revealed significant differences in the verbal fluency domain (letter fluency *p* = 0.029, d = 0.15 and category fluency *p* = 0.005, d = 0.28). A statistically significant change over time was also noted in the verbal memory domain (list learning *p* = 0.01, d = 0.36, list recall *p* = 0.003, d = 0.35, and list recognition *p* = 0.02, d = 0.40). Finally, in the domain of working memory, significant differences were noted in digit backward *p* = 0.038, d = 0.22). No statistically significant differences were seen in set-shifting, visual memory and visuospatial abilities.

**Table 2:** Neuropsychological testing raw scores by cognitive domains.

	**All participants**				
**Neuropsychological testing mean ±SD**	**Baseline** **(*n =* 52)**	**Post** **(*n =* 52)**	**95% CI**	***p*** **-value**	**|Cohens' d|**
**Verbal fluency/language**					
RBANS semantic fluency	20.6 ± 5.0	20.3 ± 4.3	−2.05, 1.24	0.63	0.06
RBANS picture naming	9.4 ± 1.0	9.6 ± 0.8	−0.11, 0.56	0.18	0.22
DKEFS letter fluency	10.9 ± 3.5	11.4 ± 3.3	0.06, 1.11	0.029 ^a^	0.15
DKEFS category fluency	12.4 ± 2.9	11.5 ± 3.4	−1.70, −0.32	0.005 ^a^^b^	0.28
**Verbal memory**					
RBANS list learning	27.0 ± 4.7	28.7 ± 4.7	3.10, 0.41	0.01^a^	0.36
RBANS list recall	5.6 ± 2.3	6.4 ± 2.3	0.32, 1.45	0.003 ^a^^b^	0.35
RBANS story memory	16.4 ± 3.6	17.3 ± 3.5	−0.27, 2.12	0.12	0.25
RBANS story recall	8.5 ± 2.5	8.9 ± 2.3	−0.43, 1.20	0.35	0.17
RBANS list recognition	18.6 ± 1.7	19.2 ± 1.3	0.09, 1.08	0.02 ^a^	0.40
**Set–shifting**					
DKEFS category switching	12.5 ± 2.8	12.7 ± 3.2	−0.89, 1.19	0.77	0.07
DKEFS category switching accuracy	12.7 ± 2.3	12.8 ± 2.9	−0.79, 0.99	0.82	0.04
**Working memory**					
Digit span forward	10.2 ± 2.4	10.4 ± 2.3	−0.30, 0.79	0.37	0.08
Digit span backward	8.8 ± 2.2	8.3 ± 2.4	−0.97, −0.03	0.038 ^a^	0.22
Digit span sequence	8.7 ± 2.2	8.4 ± 2.3	−0.81, 0.30	0.36	0.13
RBANS digit span	10.7 ± 2.2	10.8 ± 2.9	−0.65, 0.78	0.86	0.04
**Visual memory**					
RBANS figure recall	14.1 ± 2.2	14.1 ± 3.0	−0.77, 1.27	0.63	0.00
RBANS coding	43.8 ± 3.3	44.0 ± 6.5	−1.95, 2.06	0.95	0.04
**Visuospatial abilities**					
RBANS figure copy	15.8 ± 3.3	16.3 ± 2.8	−0.25, 1.35	0.17	0.16
RBANS line orientation	17.4 ± 2.4	17.2 ± 3.2	−0.92, 0.54	0.60	0.07

*RBANS, Repeatable Battery for the Assessment of Neuropsychological Status; WAIS IV, Wechsler Adult Intelligence Scale 4th Edition; DKEFS, Delis–Kaplan Executive Function System*.

a *Statistical significance (p < 0.05)*.

b *Holds FDR correction*.

### Cardiorespiratory fitness

Participants showed a pre-to-post improvement in cardiorespiratory fitness as evidenced by a statistically significant change over time in HRR1 (*p* = 0.01, d = 0.30), and HRR2 (*p* < 0.001, d = 0.50). Greater aerobic capacity over time was also found, but only in the remote participants (reps, *p* < 0.001, d = 0.43). These results can be seen in Table 3.

**Table 3:** Cardiorespiratory fitness.

**Cardiorespiratory fitness** **mean ±SD**	**Baseline** **(*n =* 52)**	**Post** **(*n =* 52)**	**95% CI**	***p*** **-value**	**Cohen's d**
**Aerobic capacity**					
In-person ^c^	501.3 ± 149.5	519.0 ± 140.7	−3.30, 38.74	0.10	0.12
Remote ^d^	26.0 ± 7.1	29.5 ± 9.2	1.69, 5.46	<0.001^a^^b^	0.43
**Heart rate recovery**					
HRR1	23.6 ± 11.6	27.0 ± 10.7	0.75, 5.98	0.01 ^a^	0.30
HRR2	33.8 ± 12.5	40.4 ± 13.8	3.24, 9.95	<0.001^a^^b^	0.50

*HRR, Heart Rate Recovery*.

a *Statistical significance (p < 0.05)*.

b *Holds FDR correction*.

c *In in-person participants fitness was measured by the total distance in meters in the Incremental Walking Shuttle Test*.

d *In remote participants fitness was measured by the total repetitions in the 1-min Sit-to-Stand Test*.

### The influence of engagement on cardiorespiratory fitness and cognition gains

Concerning the extent to which exercise engagement influenced cardiorespiratory fitness, a linear mixed-effect model revealed a *Time* effect for HRR1 (F_1, 48_= 3.68, *p* = 0.04, d = 0.56) and HRR2 (F_1, 48_= 9.73, *p* = 0.003, d = 0.91) during weeks 1–4, which demonstrates significant improvements in both outcomes, which were associated with a moderate to high effect size. In addition, there was a Time^*^Group interaction effect (F_1, 48_= 4.40, *p* = 0.041, d = 0.60) only for HHR2, also only for the first 4 weeks. This indicated that HRR2 was significantly different between the two subgroups (high and low engagement) over time, with individuals who demonstrated high engagement demonstrating greater improvements in HRR2, compared with individuals who demonstrated low engagement (Table 4). None of the remaining comparisons reached statistical significance.

**Table 4:** Exercise engagement effect on fitness.

**Fitness measure**	**Linear mixed-effect model**	**F**	***p*** **-value**	**d**
**HRR1 (bpm)**	*Time*	3.68	0.04^a^	0.56
Weeks 1–4	*Group*	3.53	0.07	0.55
	Group*Time	0.95	0.33	0.28
Weeks 5–8	*Group*	2.53	0.12	0.46
	Group*Time	1.57	0.22	0.36
Weeks 1–8	*Group*	4.17	0.047 ^a^	0.60
	Group*Time	1.08	0.30	0.30
**HRR2 (bpm)**	*Time ^b^*	9.73	0.003 ^a^^b^	0.91
Weeks 1–4	*Group*	1.70	0.20	0.38
	Group*Time	4.40	0.041^a^	0.60
Weeks 5–8	*Group*	2.53	0.12	0.46
	Group*Time	1.12	0.29	0.30
Weeks 1–8	*Group*	2.61	0.21	0.47
	Group*Time	2.13	0.15	0.43
**Aerobic capacity (z-scores)** ^d^	*Time*	-	-	
Weeks 1–4	*Group*	1.22	0.27	0.32
	Group*Time	0.19	0.66	0.12
Weeks 5–8	*Group*	9.50	0.003 ^a^^b^	0.90
	Group*Time	3.07	0.08	0.51
Weeks 1–8	*Group*	9.64	0.003 ^a^^b^	0.91
	Group*Time	1.83	0.18	0.39

*Group: (1) Low engagement, and (2) High engagement; Time: (1) Pre and (2) Post. Degrees of freedom = 1.48; MI, Moderate intensity; HI, High intensity; and MHI, Moderate-to-high intensity*.

a *Statistical significance (p < 0.05)*.

b *Holds FDR correction*.

c *No time effect was assessed in aerobic capacity due to standardized z-scores*.

d *Aerobic capacity was transformed to standardized z-scores to fit both in-person and remote fitness assessment tests*.

Concerning the extent to which exercise engagement influenced cognition, a linear mixed-effect model revealed a Group^*^Time interaction effect for the visuospatial domain (F_1, 49_= 4.34, *p* = 0.042, d = 0.61), and global cognition (F_1, 49_= 5.26, *p* = 0.026, d = 0.67). This indicated that global cognition, particularly in the visuospatial domain, was higher in participants with greater engagement (Table 5). No other comparisons reached statistical significance.

**Table 5:** Exercise engagement effect on cognition.

**Cognitive domain**	**Linear mixed-effect model**	**F**	***p*** **-value**	**d**
**Verbal fluency/language**	*Time ^b^*	-	-	
Weeks 1–8	*Group*	7.04	0.01^a^	0.77
	Group^*^Time	0.09	0.76	0.06
**Verbal memory**	*Time ^b^*	-	-	
Weeks 1–8	*Group*	2.77	0.07	0.54
	Group^*^Time	0.33	0.57	0.22
**Set-shifting**	*Time ^b^*	-	-	
Weeks 1–8	*Group*	4.71	0.034^a^	0.63
	Group^*^Time	0.36	0.54	0.18
**Working memory**	*Time ^b^*	-	-	
Weeks 1–8	*Group*	2.47	0.12	0.46
	Group^*^Time	0.20	0.66	0.13
**Visual memory**	*Time ^b^*	-	-	
Weeks 1–8	*Group*	0.71	0.40	0.25
	Group^*^Time	1.56	0.22	0.36
**Visuospatial**	*Time ^b^*	-	-	
Weeks 1–8	*Group*	0.34	0.56	0.17
	Group^*^Time	4.34	0.042^a^	0.61
**Global cognition score**	*Time ^b^*	-	-	
Weeks 1–8	*Group*	6.30	0.015^a^	0.73
	Group^*^Time	5.26	0.026^a^	0.67

*Group: (1) Low engagement, and (2) High engagement; Time: (1) Pre and (2) Post. Degrees of freedom = 1.50; MHI, Moderate-to-high intensity*.

a *Statistical significance (p < 0.05)*.

b *No time effect was assessed in cognition due to standardized z-scores*.

### Associations between cognition and fitness

The first model (Baseline) adjusted only for cognition and fitness baseline measures. The second model (Baseline + EE) adjusted for both cognition and fitness baseline, and exercise engagement (high vs. low engagement).

Concerning the association between cognitive and cardiorespiratory fitness, when controlling only for baseline performance, a multiple linear regression demonstrated higher HRR1 was associated with better visual memory (β = 0.011, d = 0.27, *p* = 0.031) and visuospatial performance (β = 0.010, d = 0.26, *p* = 0.046). Higher HRR2 was associated with worse performance in verbal fluency/language (β = −0.006, d = 0.36, *p* = 0.004) and working memory (β = −0.004, d = 0.25, *p* = 0.052), and better verbal memory (β = 0.11, d = 0.45, *p* < 0.0001). Higher aerobic capacity was associated with better working memory (β = 0.124, d = 0.32, *p* = 0.011) and a worse visuospatial performance (β = −0.251, d = 0.39, *p* = 0.002).

When additionally adjusting for exercise engagement, higher HRR1 remained significantly associated with better visual memory (β = 0.012, d = 0.32, *p* = 0.013). Higher HRR2 was associated with worse verbal fluency/language (β = −0.008, d = 0.47, *p* < 0.0001) and working memory (β = −0.005, d = 0.26, *p* = 0.041), and better verbal memory (β = 0.007, d = 0.32, *p* = 0.013). Higher aerobic capacity was associated with better working memory (β = 0.117, d = 0.3, *p* = 0.02) and worse visuospatial (β = −0.280, d = 0.45, *p* < 0.001) and visual memory performance (β = −0.196, d = 0.39, *p* = 0.011).

### Engagement: Time spent in prescribed heart rate zone

Participants were instructed to spend 100% of intervention time (total time = 24 h) within their prescribed target heart rate zone. Despite being supervised for the total time, participants spent 76.7% of the total intervention time in the prescribed target heart rate zone. Further, they demonstrated high engagement 63.4% of the time and low-to-moderate engagement 34.6% of the time. Because engagement was found to significantly modify the cardiorespiratory and cognitive effects, we conducted exploratory analyses to assess if engagement differed between in-person and remote participants. Surprisingly, in-person and remote participants demonstrated differences in exercise engagement during the intervention (*p* < 0.001, d = 1.19), with the remote participants demonstrating higher exercise engagement. Details are provided in Table 6.

**Table 6:** Exercise engagement.

**Exercise Engagement, time spent in prescribed target heart rate zone**	**All participants (*n =* 52)**	**In-person** **(*n =* 19)**	**Remote** **(*n =* 33)**	**95% CI**	***p*** **-value**	**Cohen's d**
**Weeks 1–8**, mean (95% CI)	76.7 (73.9, 79.6)	61.8 (58.5, 65.0)	85.4 (81.9, 88.9)	−35.3, −11.9	<0.001^a^^b^	1.19
Low-to-moderate, *n* (%)	18 (34.6)	12 (63.2)	6 (18.2)			
High, *n* (%)	34 (65.4)	7 (36.8)	27 (81.8)			

*Exercise engagement: Low-to-moderate <69.9%, High > 70.0%*.

a *Statistical significance (p < 0.05)*.

b *Holds FDR correction*.

## Discussion

In our study, after 8 weeks of supervised moderate-to-high-intensity aerobic exercise, participants demonstrated statistically significant improvements of a moderate effect in both HRR1 and HRR2. Verbal fluency/language and verbal memory showed statistically significant improvements, and working memory showed a statistically significant decrement, but these were associated with a small effect size. Exercise engagement, or the time spent in the prescribed heart rate zone, significantly influenced both improvements in cardiorespiratory fitness and cognitive performance regardless of whether the intervention was completed in the laboratory with in-person supervision or at home with remote supervision. An exploratory analysis revealed that remote participants were more likely to have higher exercise engagement.

Gains in cognitive performance and cardiorespiratory fitness were significantly associated with various cognitive domains, but these associations were associated with small effect sizes. The main objective of the present study was to identify clinically meaningful effects that are relevant for clinical practice and research efforts aimed at optimizing exercise interventions for cognitive gains in aging. For this reason, this discussion will mainly focus on statistically significant findings that reached at least a moderate effect size, and thus may have clinically meaningful implications.

Cardiorespiratory fitness improvements are typically measured in estimated oxygen consumption, defined as the gold-standard measure of the body's efficiency to intake, circulate and utilize oxygen during incremental exercise (40, 41). In the present study, we measured changes in cardiorespiratory function with exercise tests that are mostly used in clinical practice. HRR measures the autonomic regulation involved in the cessation of exercise, with HRR1 being predominantly attributed to an increase in parasympathetic activity, and HRR2 additionally being attributed to sympathetic decrease (26). Importantly, unlike the assessment of maximal oxygen consumption that requires over an hour to administer and access to sophisticated equipment, HRR can be measured in a few minutes requiring only access to a heart rate monitor, and minimal training. Given that exercise is more effective than any therapeutic that currently exists to maintain and improve cognitive brain health, and that cognitive improvements are driven by cardiorespiratory gains, access to simple measures like HRR will enable refining, optimization, and individualization of exercise interventions for cognitive brain health in aging and older adults.

While it is generally accepted that the minimum amount of time that is considered necessary for exercise to promote measurable improvements in cardiorespiratory performance is approximately 3 months (42), our exercise program length is consistent with other studies that have demonstrated 8 weeks to be sufficient to show positive changes in cardiorespiratory fitness and function (43, 44). Our findings of an improvement in HRR in as little as 4 weeks of moderate intensity exercise are encouraging and suggest that HRR may capture cardiorespiratory improvements that are relevant for exercise-induced cognitive gains.

A few interpretations exist for the discrepancy in the relationship between the HRR and aerobic capacity measures and the cognitive outcomes in the present study. It is likely that the use of indirect measures of aerobic capacity (total distance in the incremental shuttle and total repetitions on the 1-min sit to stand) contributed to the lack of associations with the cognitive outcomes. Another consideration is that the exercise dose (both in overall time and in group-level effort) could have been insufficient, and that a longer dose (exposure) of exercise could have been required to promote greater improvements in aerobic capacity.

Consistent with prior studies, we observed that some, but not all cognitive domains showed significant changes from baseline following the 8-week exercise program (5, 45–49). Additional support for the fact that we may have been underdosed in our exercise dose was that improvements in verbal fluency/language and verbal memory did not reach at least a moderate effect size. For the same reason, the results of the multiple regression are likely less clinically meaningful as well. While it is also possible that these changes may be attributed to a practice effect, alternate versions of each test (except the digit span) were used pre-post to mitigate this effect. However, the examination of practice effects in itself is becoming increasingly more important in the brain health interventional literature, given that the absence of a practice effect may actually be an early indicator of cognitive impairment (50).

The performance decrement seen in working memory is most likely due to test variability (this domain was assessed with components of the digit span, which was not administered with an alternate version), and given the small effect size, we do not believe this was clinically meaningful. Although our data do not allow us to differentiate between worsening of working memory due to test variability vs. a deleterious effect of the intervention on working memory or a natural progression of cognitive decline in that domain, we feel the latter two explanations are unlikely. First, our study participants were individuals who had been screened for cognitive impairment prior to study participation and in whom there was no evidence of pre-existing cognitive impairment or neurodegenerative disease. Therefore, natural progression of cognitive decline seems unlikely. Second, natural progression of cognitive decline specific to working memory in only 2 months in individuals who at baseline are cognitively unimpaired seems also most unlikely. Third, a specific deleterious effect of the intervention on working memory and in only a 2 month intervention, seems highly unlikely. Thus, we feel the most sensible explanation for the unexpected findings is in fact test variability, i.e. that the working memory “worsening” is in fact artifactual. It is also worth noting that future studies with greater exercise dosages should be conducted to replicate these results, and additionally perform comparisons with active control groups.

We found that exercise engagement was a meaningful indicator of the extent to which individuals made gains in both cardiorespiratory and cognitive function. Individuals with higher engagement also had greater improvements in HRR2 during the first 4 weeks, and greater gains in global cognition, particularly in the visuospatial domain. While not surprising, we believe that this finding has important clinical implications given that exercise engagement is rarely measured in most clinical trials of exercise and cognition (7). The broader implication of this finding is the need to include measures of effort, motivation, and engagement in clinical practice and clinical research in this field given that our findings suggest they may be driving improvements in cardiorespiratory and cognitive function.

Necessary adjustments to continue study enrollment during restrictions imposed by the COVID pandemic resulted in the fact that participants used slightly different aerobic stimuli to reach their prescribed heart rate zones. In-person participants completed their exercise on a bike, treadmill or elliptical, while remote participants completed alternating body weight exercises. While it is important to note that participants received the same instructions, and were monitored in the same manner, our analyses demonstrated that the groups differed in adherence and also in exercise engagement. First, study adherence was much higher during remote participation of the study (97.1% remote vs. 46.3% in-person). This may be attributed to the removal of barriers to exercise, such as travel time and expenses, gym access, and allowing individuals the flexibility to do it on their own time.

It is possible that the better outcomes in adherence and engagement in remote participants could have been influenced by the lack of social mobility and interaction during that time, which would make our participants especially motivated to meet with the study investigators and participate in the sessions. However, a study sought to understand how this shift to remotely supervised exercises during the COVID-19 pandemic affected participation and study adherence, and interestingly found no difference in attendance of exercise sessions between in-person and remote participants (51). Greater adherence and greater engagement in remote exercises suggests that at-home exercise programs may be beneficial for aging sedentary adults who find it difficult to maintain an exercise program through a traditional gym. Further studies are warranted to understand the meaningfulness of increased adherence and engagement to remote exercise programs.

The study population was considered at risk of cognitive decline due to health and lifestyle factors described in Table 1, age, and because they were sedentary older adults. In this study the average age of the sample was 62.9, with the youngest participant being 56. As age is the biggest risk factor for dementia, all study participants are at risk due to their age alone. All subjects in this study were sedentary (as determined by the short version of the International Physical Activity Questionnaire [IPAQ]). Physical inactivity is a well-studied risk factor for dementia. Additional factors in the study population contributing to risk of dementia include smoking (25% of study population), hypertension (48.1%), depression/anxiety (25%), heart disease (15.4%), diabetes (7.7%), and hearing loss (11.5%) (4, 52–54). The in-person and remote groups were demographically similar, with the exception of ethnicity. The in-person participants were largely Hispanic, while the remote participants were largely non-Hispanic Whites. We also observed more women participants in the remote group, although this was not statistically significant. It is important to note that recruitment methods were altered to accommodate the switch to a remote protocol after the onset of the COVID-19 pandemic, and the ethnic differences may relate to the changes in recruitment. In-person participants were primarily recruited from flyers posted on the University of Miami Miller campus and in public libraries. After the pandemic began, recruitment was moved to virtual platforms, including social media and Nextdoor.com. The difference in ethnicity in in-person and remote participants is likely related to differences in the populations who utilize these resources, such as socioeconomic and health status. This resulted in an overall sample that is likely skewed toward women with higher socioeconomic and better health status, and more technology savvy who could have reliable access to an intervention delivered *via* telehealth to successfully operate zoom. Given the findings obtained, future studies exploring race/ethnicity effects seem important and it will be critical to separate those from specific cultural or socio-economic factors.

The lack of a control group can be seen as a limitation, especially with neuropsychological performance measures that are influenced by practice effects (50). However, we carefully considered several potential study designs in the planning of the present investigation and decided that a single-arm intervention trial would be most appropriate to address our study aims. Thus, we did not include a control group, nor did we blind raters to the exercise that participants were undertaking due to logistical and resource availability. We were most interested in why some people show greater cognitive benefits to aerobic exercise than others do. This is a much different question than if on a group level, a sample improves in cognitive performance after exercising relative to a control group, a finding that is well established (4). Our study was planned as a first approximation to identify preliminary effects that could then be tested in a future controlled study. We did not stratify participants by cognitive improvement, as that was beyond the scope of the present study. We did however stratify participants by exercise engagement, which is reported to be a driver of change in fitness, which in turn may promote changes in cognition. Regardless of exercise mode, individuals received the same instructions, as the focus was always to achieve and attempt to maintain the target heart rate zone. Future studies with adequate sample sizes should further examine such questions.

While the use of technology can mitigate some barriers, it can also raise barriers especially for those of disadvantaged backgrounds. Future exercise programs to promote brain health should be inclusive, and thus, future research will be necessary to develop and test exercise interventions that will be inclusive on the basis of gender, education levels, health status, socioeconomic status and digital literacy, among other factors. As mentioned before, it is likely that more robust results would have been seen with a larger sample and a longer intervention. Finally, it would have been very interesting to have a post-study follow-up period to assess if individuals had been successful in continuing to exercise regularly.

## Conclusions

The results of this study suggest that 8 weeks of moderate to high-intensity aerobic exercise can sufficiently increase cardiorespiratory fitness and improve cognitive function. This study also suggests that observed improvements may be driven by the changes in exercise engagement. This link between aerobic exercise driving improvements in cardiorespiratory fitness and cognitive function is of great importance for sedentary, aging individuals who are at risk of age-related cognitive decline.

## Data availability statement

The original contributions presented in the study are included in the article/supplementary materials, further inquiries can be directed to the corresponding author/s.

## Ethics statement

The studies involving human participants were reviewed and approved by the Institutional Review Board at the University of Miami Miller School of Medicine. The patients/participants provided their written informed consent to participate in this study.

## Author contributions

JG-O, TR, AP-L, and DL participated in the conception and design of the study. LC participated as a consultant. DC, CN, JR, MF, and CH participated in different aspects related to the collection and organization of data. DC, JG-O, and GC contributed to the statistical analysis and interpretation of the results. CH, MC, DC, and JG-O contributed to the writing of the first draft of the manuscript. All authors contributed to manuscript revision, read, and approved the submitted version.

## Funding

JG-O was supported by an Evelyn F. McKnight Pilot Grant. The project described was supported by the National Center for Advancing Translational Sciences of the National Institutes of Health under Award Number KL2TR002737.

## Conflict of interest

Authors MC, AP-L, and JG-O were employed by Linus Health. The remaining authors declare that the research was conducted in the absence of any commercial or financial relationships that could be construed as a potential conflict of interest.

## Publisher's note

All claims expressed in this article are solely those of the authors and do not necessarily represent those of their affiliated organizations, or those of the publisher, the editors and the reviewers. Any product that may be evaluated in this article, or claim that may be made by its manufacturer, is not guaranteed or endorsed by the publisher.

## Author's disclaimer

The content is solely the responsibility of the authors and does not necessarily represent the official views of the National Institutes of Health.

## References

[1] Cummings J Lee G Mortsdorf T Ritter A Zhong K. Alzheimer's disease drug development pipeline: 2017. Alzheimers Dement N Y N. (2017) 3:367–84. 10.1016/j.trci.2017.05.002 29067343 PMC5651419

[2] Cummings JL Morstorf T Zhong K. Alzheimer's disease drug-development pipeline: few candidates, frequent failures. Alzheimers Res Ther. (2014) 6:37. 10.1186/alzrt269 25024750 PMC4095696

[3] Decade of Healthy Ageing (2021–2030) [Internet]. Available online at: https://www.who.int/initiatives/decade-of-healthy-ageing (accessed April 7, 2022)

[4] Livingston G Huntley J Sommerlad A Ames D Ballard C Banerjee S. Dementia prevention, intervention, and care: 2020 report of the Lancet Commission. Lancet. (2020) 396:413–46. 10.1016/S0140-6736(20)30367-6 32738937 PMC7392084

[5] Colcombe S Kramer AF. Fitness effects on the cognitive function of older adults: a meta-analytic study. Psychol Sci. (2003) 14:125–30. 10.1111/1467-9280.t01-1-01430 12661673

[6] Etnier JL Nowell PM Landers DM Sibley BA, A. meta-regression to examine the relationship between aerobic fitness and cognitive performance. Brain Res Rev. (2006) 52:119–30. 10.1016/j.brainresrev.2006.01.002 16490256

[7] Gomes-Osman J Cabral DF Morris TP McInerney K Cahalin LP Rundek T. Exercise for cognitive brain health in aging. Neurol Clin Pract. (2018) 8:257–65. 10.1212/CPJ.0000000000000460 30105166 PMC6075983

[8] Smith PJ Blumenthal JA Hoffman BM Cooper H Strauman TA Welsh-Bohmer K. Aerobic Exercise and Neurocognitive Performance: a Meta-Analytic Review of Randomized Controlled Trials. Psychosom Med. (2010) 72:239–52. 10.1097/PSY.0b013e3181d14633 20223924 PMC2897704

[9] McPhee JS French DP Jackson D Nazroo J Pendleton N Degens H. Physical activity in older age: perspectives for healthy ageing and frailty. Biogerontology. (2016) 17:567–80. 10.1007/s10522-016-9641-0 26936444 PMC4889622

[10] Erickson KI Hillman C Stillman CM Ballard RM Bloodgood B Conroy DE. Physical activity, cognition, and brain outcomes: a review of the 2018 physical activity guidelines. Med Sci Sports Exerc. (2019) 51:1242–51. 10.1249/MSS.0000000000001936 31095081 PMC6527141

[11] Zubala A MacGillivray S Frost H Kroll T Skelton DA Gavine A. Promotion of physical activity interventions for community dwelling older adults: a systematic review of reviews. PLoS ONE. (2017) 12:e0180902. 10.1371/journal.pone.0180902 28700754 PMC5507305

[12] Pascual-Leone A. To reduce the risk of dementia, focus on the patient. Ann Neurol. (2021) 89:1080–3. 10.1002/ana.26086 33866586

[13] Chen FT Etnier JL Chan KH Chiu PK Hung TM Chang YK. Effects of Exercise Training Interventions on Executive Function in Older Adults: A Systematic Review and Meta-Analysis. Sports Med Auckl Nz. (2020) 50:1451–67. 10.1007/s40279-020-01292-x 32447717 PMC7376513

[14] Northey JM Cherbuin N Pumpa KL Smee DJ Rattray B. Exercise interventions for cognitive function in adults older than 50: a systematic review with meta-analysis. Br J Sports Med. (2018) 52:154–60. 10.1136/bjsports-2016-096587 28438770

[15] Guadagni V Drogos LL Tyndall AV Davenport MH Anderson TJ Eskes GA. Aerobic exercise improves cognition and cerebrovascular regulation in older adults. Neurology. (2020) 94:e2245–57. 10.1212/WNL.0000000000009478 32404355 PMC7357295

[16] Yu JT Xu W Tan CC Andrieu S Suckling J Evangelou E. Evidence-based prevention of Alzheimer's disease: systematic review and meta-analysis of 243 observational prospective studies and 153 randomised controlled trials. J Neurol Neurosurg Psychiatry. (2020) 91:1201–9. 10.1136/jnnp-2019-321913 32690803 PMC7569385

[17] Dustman RE Ruhling RO Russell EM Shearer DE Bonekat HW Shigeoka JW. Aerobic exercise training and improved neuropsychological function of older individuals. Neurobiol Aging. (1984) 5:35–42. 10.1016/0197-4580(84)90083-6 6738784

[18] Opel N Martin S Meinert S Redlich R Enneking V Richter M. White matter microstructure mediates the association between physical fitness and cognition in healthy, young adults. Sci Rep. (2019) 9:12885. 10.1038/s41598-019-49301-y 31501448 PMC6733843

[19] Stigger FS Zago Marcolino MA. Portela KM, Plentz RDM. Effects of exercise on inflammatory, oxidative, and neurotrophic biomarkers on cognitively impaired individuals diagnosed with dementia or mild cognitive impairment: a systematic review and meta-analysis. J Gerontol Ser A. (2019) 74:616–24. 10.1093/gerona/gly173 30084942

[20] Wittfeld K Jochem C Dörr M Schminke U Gläser S Bahls M. Cardiorespiratory fitness and gray matter volume in the temporal, frontal, and cerebellar regions in the general population. Mayo Clin Proc. (2020) 95:44–56. 10.1016/j.mayocp.2019.05.030 31902428

[21] Christie BR Swann SE Fox CJ Froc D Lieblich SE Redila V. Voluntary exercise rescues deficits in spatial memory and long-term potentiation in prenatal ethanol-exposed male rats. Eur J Neurosci. (2005) 21:1719–26. 10.1111/j.1460-9568.2005.04004.x 15845099

[22] Christie BR Eadie BD Kannangara TS Robillard JM Shin J Titterness AK. Exercising our brains: how physical activity impacts synaptic plasticity in the dentate gyrus. Neuromolecular Med. (2008) 10:47–58. 10.1007/s12017-008-8033-2 18535925

[23] van Praag H. Neurogenesis and exercise: past and future directions. Neuromolecular Med. (2008) 10:128–40. 10.1007/s12017-008-8028-z 18286389

[24] Garber CE Blissmer B Deschenes MR Franklin BA Lamonte MJ Lee IM. Quantity and quality of exercise for developing and maintaining cardiorespiratory, musculoskeletal, and neuromotor fitness in apparently healthy adults: guidance for prescribing exercise. Med Sci Sports Exerc. (2011) 43:1334–59. 10.1249/MSS.0b013e318213fefb 21694556

[25] Pentikäinen H Savonen K Ngandu T Solomon A Komulainen P Paajanen T. Cardiorespiratory fitness and cognition: longitudinal associations in the FINGER study. J Alzheimers Dis JAD. (2019) 68:961–8. 10.3233/JAD-180897 30814346

[26] Pierpont GL Adabag S Yannopoulos D. Pathophysiology of exercise heart rate recovery: a comprehensive analysis. Ann Noninvasive Electrocardiol. (2013) 18:107–17. 10.1111/anec.12061 23530480 PMC6932299

[27] Cole CR Blackstone EH Pashkow FJ Snader CE Lauer MS. Heart-rate recovery immediately after exercise as a predictor of mortality. N Engl J Med. (1999) 341:1351–7. 10.1056/NEJM199910283411804 10536127

[28] Intzandt B Sabra D Foster C Desjardins-Crépeau L Hoge RD Steele CJ. Higher cardiovascular fitness level is associated with lower cerebrovascular reactivity and perfusion in healthy older adults. J Cereb Blood Flow Metab. (2020) 40:1468–81. 10.1177/0271678X19862873 31342831 PMC7308519

[29] Qiu S Cai X Sun Z Li L Zuegel M Steinacker JM. Heart rate recovery and risk of cardiovascular events and all-cause mortality: a meta-analysis of prospective cohort studies. J Am Heart Assoc Cardiovasc Cerebrovasc Dis. (2017) 6:e005505. 10.1161/JAHA.117.005505 28487388 PMC5524096

[30] Cabral DF Hinchman CA Nunez C Rice J Loewenstein DA Cahalin LP. Harnessing neuroplasticity to promote brain health in aging adults: protocol for the MOVE-cog intervention study. JMIR Res Protoc. (2021) 10:e33589. 10.2196/33589 34817393 PMC8663452

[31] Randolph C Tierney MC Mohr E Chase TN. The repeatable battery for the assessment of neuropsychological status (RBANS): preliminary clinical validity. J Clin Exp Neuropsychol. (1998) 20:310–9. 10.1076/jcen.20.3.310.823 9845158

[32] Acevedo A Loewenstein DA Barker WW Harwood DG Luis C Bravo M. Category fluency test: normative data for English- and Spanish-speaking elderly. J Int Neuropsychol Soc JINS. (2000) 6:760–9. 10.1017/S1355617700677032 11105466

[33] LaBelle DR Lee BG Miller JB. Dissociation of executive and attentional elements of the digit span task in a population of older adults: a latent class analysis. Assessment. (2019) 26:1386–98. 10.1177/1073191117714556 28621146

[34] Dourado VZ Vidotto MC Guerra RLF. Reference equations for the performance of healthy adults on field walking tests. J Bras Pneumol Publicacao Soc Bras Pneumol E Tisilogia. (2011) 37:607–14. 10.1590/S1806-37132011000500007 22042392

[35] Singh SJ Morgan MD Scott S Walters D Hardman AE. Development of a shuttle walking test of disability in patients with chronic airways obstruction. Thorax. (1992) 47:1019–24. 10.1136/thx.47.12.1019 1494764 PMC1021093

[36] Bohannon RW Crouch R. 1-Minute sit-to-stand test: systematic review of procedures, performance, and clinimetric properties. J Cardiopulm Rehabil Prev. (2019) 39:2–8. 10.1097/HCR.0000000000000336 30489442

[37] Borg GA. Psychophysical bases of perceived exertion. Med Sci Sports Exerc. (1982) 14:377–81. 10.1249/00005768-198205000-00012 7154893

[38] Lee SY Jennrich RI. A study of algorithms for covariance structure analysis with specific comparisons using factor analysis. Psychometrika. (1979) 44:99–113. 10.1007/BF02293789

[39] Cohen J. Statistical Power Analysis for the Behavioral Sciences. Cambridge: Academic Press. (1977).

[40] ACSM's Health-Related Physical Fitness Assessment Manual. Fourth. Lippincott Williams & Wilkins (2013).

[41] Wasserman K Van Kessel AL Burton GG. Interaction of physiological mechanisms during exercise. J Appl Physiol. (1967) 22:71–85. 10.1152/jappl.1967.22.1.71 6017656

[42] Bacon AP Carter RE Ogle EA Joyner MJ. VO2max trainability and high intensity interval training in humans: a meta-analysis. PLoS ONE. (2013) 8:e73182. 10.1371/journal.pone.0073182 24066036 PMC3774727

[43] Bruseghini P Calabria E Tam E Milanese C Oliboni E Pezzato A. Effects of 8 weeks of aerobic interval training and of isoinertial resistance training on risk factors of cardiometabolic diseases and exercise capacity in healthy elderly subjects. Oncotarget. (2015) 6:16998–7015. 10.18632/oncotarget.4031 26046575 PMC4627287

[44] Omidi S Hosseinpour Delavar S Amiripour A Heydarpoor B. Effects of 8 weeks of aerobic exercises on the cardiac function and inflammatory markers of male patients with heart failure after coronary artery bypass grafting. J Kermanshah Univ Med Sci. (2020) 24. 10.5812/jkums.98429

[45] Angevaren M Aufdemkampe G Verhaar HJJ Aleman A Vanhees L. Physical activity and enhanced fitness to improvecognitive function in older people without known cognitive impairment. Cochrane Database Syst Rev. (2008) 3:CD005381. 10.1002/14651858.CD005381.pub2 18646126

[46] Kelly ME Loughrey D Lawlor BA Robertson IH Walsh C Brennan S. The impact of exercise on the cognitive functioning of healthy older adults: a systematic review and meta-analysis. Ageing Res Rev. (2014) 16:12–31. 10.1016/j.arr.2014.05.002 24862109

[47] Ludyga S Gerber M Brand S Holsboer-Trachsler E Pühse U. Acute effects of moderate aerobic exercise on specific aspects of executive function in different age and fitness groups: a meta-analysis. Psychophysiology. (2016) 53:1611–26. 10.1111/psyp.12736 27556572

[48] Wayne PM Walsh JN Taylor-Piliae RE Wells RE Papp KV Donovan NJ. Effect of tai chi on cognitive performance in older adults: systematic review and meta-analysis. J Am Geriatr Soc. (2014) 62:25–39. 10.1111/jgs.12611 24383523 PMC4055508

[49] Young J Angevaren M Rusted J Tabet N. Aerobic exercise to improve cognitive function in older people without known cognitive impairment. Cochrane Database Syst Rev. (2015) 4:CD005381. 10.1002/14651858.CD005381.pub4 25900537 PMC10554155

[50] Jutten RJ Rentz DM Fu JF Mayblyum DV Amariglio RE Buckley RF. Monthly at-home computerized cognitive testing to detect diminished practice effects in preclinical Alzheimer's disease. Front Aging Neurosci. (2022) 13:800126. 10.3389/fnagi.2021.800126 35095476 PMC8792465

[51] García Pérez de. Sevilla G Barceló Guido O De la Cruz M de la P Fernández AB Alejo LB Ramírez Goercke MI. Long-term adherence to a healthy lifestyle remotely supervised exercise during the COVID-19 pandemic versus in-person-supervised exercise in achieving. Int J Environ Res Public Health. (2021) 18:12198. 10.3390/ijerph182212198 34831954 PMC8619241

[52] Kivipelto M Ngandu T Laatikainen T Winblad B Soininen H Tuomilehto J. Risk score for the prediction of dementia risk in 20 years among middle aged people: a longitudinal, population-based study. Lancet Neurol. (2006) 5:735–41. 10.1016/S1474-4422(06)70537-3 16914401

[53] Nedelec T Couvy-Duchesne B Monnet F Daly T Ansart M Gantzer L. Identifying health conditions associated with Alzheimer's disease up to 15 years before diagnosis: an agnostic study of French and British health records. Lancet Digit Health. (2022) 4:e169–78. 10.1016/S2589-7500(21)00275-2 35216751

[54] Schiepers OJG Köhler S Deckers K Irving K O'Donnell CA van den Akker M. Lifestyle for Brain Health (LIBRA): a new model for dementia prevention. Int J Geriatr Psychiatry. (2018) 33:167–75. 10.1002/gps.4700 28247500

